# A gradient relationship between low birth weight and IQ: A meta-analysis

**DOI:** 10.1038/s41598-017-18234-9

**Published:** 2017-12-21

**Authors:** Huaiting Gu, Lixia Wang, Lingfei Liu, Xiu Luo, Jia Wang, Fang Hou, Pauline Denis Nkomola, Jing Li, Genyi Liu, Heng Meng, Jiajia Zhang, Ranran Song

**Affiliations:** 1 Department of Maternal and Child Health, and MOE (Ministry of Education) Key Laboratory of Environment and Health, School of Public Health, Tongji Medical College, Huazhong University of Science and Technology, Wuhan, 430030 China; 2 School of Public Health, Jining Medical College, Jining, 272067 China; 3 Department of Radiology, Union Hospital, Tongji Medical College, Huazhong University of Science and Technology, Wuhan, 430030 China; 4 Department of Epidemiology and Biostatistics, Arnold School of Public Health, University of South Carolina, Columbia, 29208 USA

## Abstract

Multiple studies have reported that individuals with low birth weights (LBW, <2500 g) have a lower intelligence quotient (IQ) than those with normal birth weights (NBW, ≥2500 g). Based on 57 eligible individual studies including 12,137 participants, we performed a meta-analysis to estimate the association between low birth weight and individuals’ IQ scores (IQs). The pooled weight mean difference (WMD) in IQs between NBW and LBW individuals was 10 (95% CI 9.26–11.68). The WMD was stable regardless of age. No publication bias was detected. The mean IQs of the extremely low birth weight (ELBW, <1000 g), very low birth weight (VLBW, 1000–1499 g), moderately low birth weight (MLBW, 1500–2499 g) and NBW individuals were 91, 94, 99 and 104, respectively. Additionally, the WMD in IQs with NBW were 14, 10 and 7 for ELBW, VLBW, and MLBW individuals, respectively. Two studies permitted estimates of the influence of social determinants of health to the discrepancy in IQs, which was 13%. Since IQ is inherited and influenced by environmental factors, parental IQs and other factors contribute to residual confounding of the results. As the conclusion was based on population studies, it may not be applicable to a single individual.

## Introduction

Infants with low birth weight (LBW), very low birth weight (VLBW) and extremely low birth weight (ELBW) are considered to be at a high risk of cognitive dysfunction^1–3^, such as attention deficit^4,5^, executive function issues^6–8^ and low average to borderline intelligence quotient (IQ)^1,4,6–8^. With the development of perinatal care and neonatal medicine, the survival rates of LBW infants are greatly improved^5^, followed by an increasing number of LBW individuals with cognitive deficit^2,9^, which has become a serious public health burden^5,10^.

Numerous studies have focused on the cognitive outcomes of VLBW individuals in recent decades^11^. More than 50% of VLBW children required special education services, and approximately 20% of VLBW children repeated at least one grade^12^. ELBW individuals without major disabilities (mental retardation, cerebral palsy, deafness, or blindness)^5^ had subtle neurodevelopmental disabilities (language disorders, hyperactivity, behavioural problems, or motor dysfunction, etc.) in the school and teenage years^13,14^. Evidence from cohort studies in four western countries showed that more than 50% of adolescents with ELBW had learning difficulties (mathematics, writing, reading, or spelling)^15,16^. The effect of LBW accounted for a 0.4 standard deviation (SD) decrease in math and a 0.25 SD decrement in reading^17^. Those cognitive disadvantages would lead to low school achievements and persist into early adulthood^18–21^, thus resulting in low socio-economic status (SES) in the future^3^.

The IQ score (IQs) is often used to indicate individuals’ cognitive outcomes worldwide^22^. The IQ is relatively stable and can be easily measured^23^. Additionally, there are some internationally recognized assessment scales which make it possible to compare the IQs in different populations. The consistent finding was that LBW individuals had lower IQs than those with normal birth weights (NBW)^8,9,24^. The size of this discrepancy varied across studies, ranging from 3 to 23 points^9,25^, and the discrepancy was directly proportional to their birth weight^20^ (R^2^ = 0.51; *P* < 0.001)^2^. Some studies found that a gradient relationship existed, in which lower birth weight was associated with lower IQs^1,26^. In other words, the ELBW individuals’ IQs were the lowest, followed by those with VLBW and moderately low birth weight (MLBW)^27,28^. However, most of the previous individual studies were based on a small number of participants, so it was necessary to use meta-analysis to enlarge the sample size and assess the gradient relationship.

A recent meta-analysis containing 15 individual studies on the relationship between LBW and IQs in adolescent and early adulthood (age ≥ 13)^29^ found that LBW individuals scored an average of 8 IQ points lower than NBW individuals. As is already known, there have been more relevant studies focusing on preschool and school-aged children. We integrated those studies into our meta-analysis to identify the age-related change in IQs between LBW and NBW individuals.

Data from the US Centres for Disease Control showed that 45% of babies born preterm were < 2500 g^29^. Using 27 eligible individual studies published between 1980–2009, Kerr-Wilson *et al*.^30^ performed a meta-analysis on preterm delivery and intelligence, which showed that the preterm children had significantly lower IQs compared with term children. The weighted mean difference (WMD) was 12 [95% confidence interval (CI) 10.47–13.42]. The group’s analysis included duplicated populations (Caldú^31^, Narberhaus^32^), and some control groups were used more than once in the model, which may enlarge the weight of some individual studies. Despite the overlap of LBW and prematurity, they may have different relationships with IQs^29^. To more specifically reflect on the relationship between LBW and IQs, we performed this meta-analysis on LBW and IQs.

In this meta-analysis, we aimed to use 57 eligible individual studies to estimate the pooled discrepancy in IQs between LBW individuals and NBW individuals and the changes in discrepancy across age. We also used subgroup analysis to assess the gradient relationship with IQs for the different levels of LBW.

## Results

### Search results

**Figure 1:**
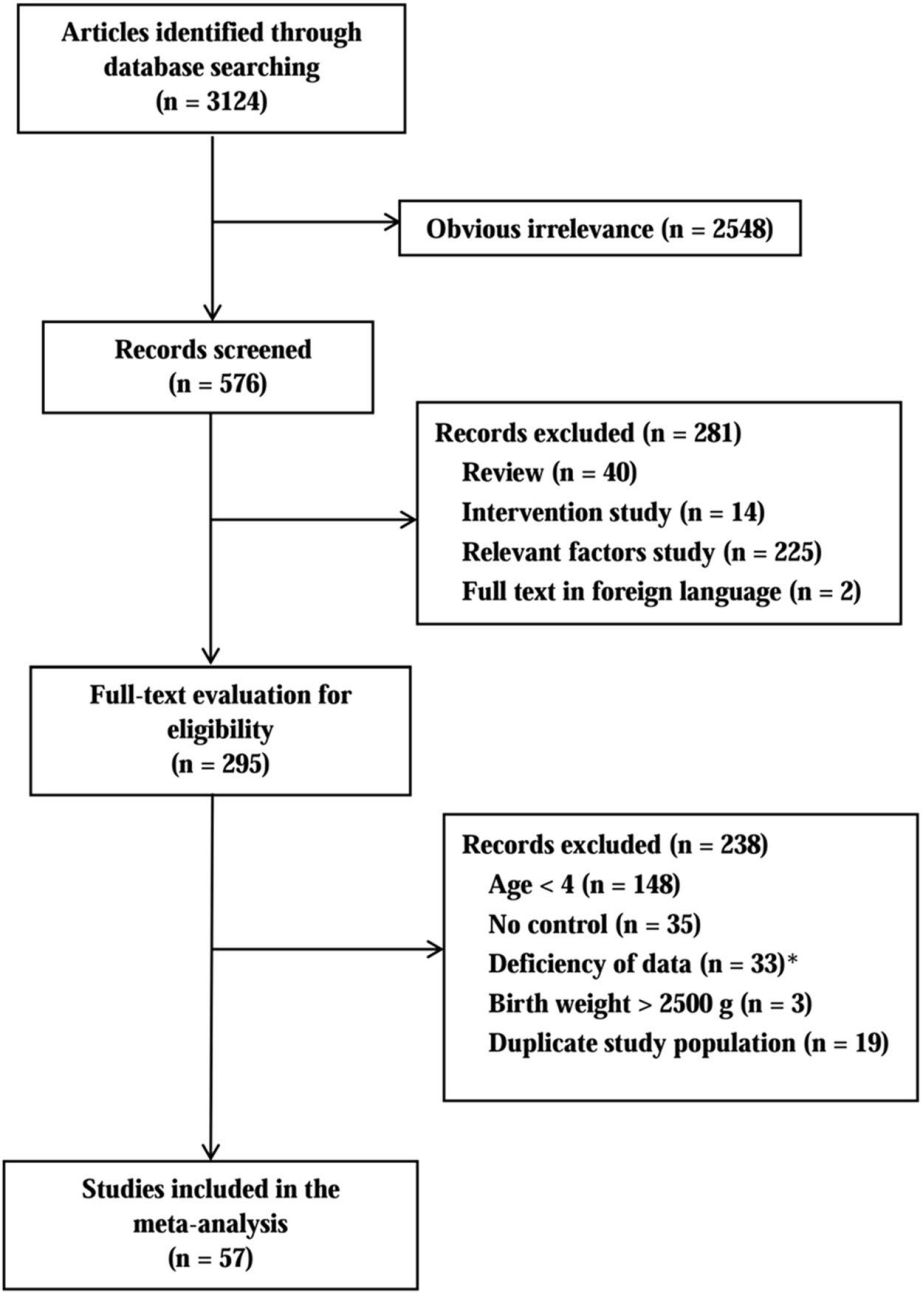
Flow chart of meta-analysis for exclusion/inclusion of individual studies. ∗Deficiency of data cited references^63–95^.

The search strategy generated a total of 3,124 potentially relevant papers. After reviewing the title and abstract, 2,548 papers were excluded because of irrelevance. Another 281 articles were also excluded because they were reviews (n = 40) or intervention studies (n = 14). Furthermore, 225 studies focusing on relevant factors for LBW and 2 studies in other than English were also excluded. Thus, we reviewed 295 articles with full text. Among them, 238 were excluded because they did not meet the inclusion criteria. The flow chart for exclusion/inclusion of individual studies is presented in Fig. 1.

### Characteristics of included studies

There were 57 eligible studies published over 36 years based on our search strategies, four of which^13,25,33,34^ had two pairs of groups in the study population. Therefore, the meta-analysis included 61 study groups with 6,683 LBW individuals and 5,454 NBW comparisons. The participants included both children and adults, with ages ranging from 4 to 26. These studies were performed in 21 countries, including 18 developed countries, where most of the studies were conducted (n = 53). Forty-four studies used different versions of the Wechsler scale to measure IQ. Five studies used the K-ABC (Kaufman Assessment Battery for Children), whereas three used the Stanford-Binet intelligence scale. The MIQS (McCarthy IQ Scale) and BAS (British Ability Scales) were used in other studies. Most studies (n = 50) were cohort studies, and 7 were case-control studies. The descriptive information of the included studies is shown in Table 1.

**Table 1:** Characteristics of included studies of LBW and IQ.

No.	Study	Country	Years of Birth	LBW(n)		Birth Weight (g)	Gestational Age (week)	Measurement Tool	Age at evaluation	IQ Scores	Study Design
				NBW(n)		mean (SD/range)	Mean (SD)/range			mean (SD)	
1	Yi KH *et al*.^96^, 2016	Korea	2003-2009	L	46	2110(315)	≥37	WISC-III	12	100.52(15.24)	case-control study
				N	46	3280(460)	≥37			109.52(12.54)	
2	Serenius *et al*.^10^, 2016	Sweden	2004–2007	L	371	779(170)	25.4(1.07)	WISC-IV	6.5	83.4(14.8)	cohort study
				N	367	3617(482)	39.9(1.13)			100.3(11.7)	
3	Breeman *et al*.^45^, 2015	German	1985–1986	L3	216	1311(320)	<32	WISC-III	26	86.2(20.25)	cohort study
				N3	197	3371(452)	>37			102.6(12.89)	
4	Molloy *et al*.^97^, 2014	Australia	1994–1995	L	221	883(161)	26.6(2.0)	WASI	18	95.18 (16.33)	cohort study
				N	159	3394(454)	39.2(1.4)			106.46(13.72)	
5	Ritter *et al*.^8^, 2014	Switzerland	1998–2003	L	52	<1500	<32	WISC (HAWIK-IV)		101.17(10.34)	cohort study
				N	36	>2500	>37		10	109.28(7.77)	
6	Guarini *et al*.^25^, 2014	Italy	1998–2001	L1	56	1155(331)	29.8(2.3)	K-BIT	6	93.4(10.5)	case-control study
				N1	60	>2800	>37			96.7(11.4)	
			1996–1999	L2	84	1224(284)	30.1(2.3)		8	103.1(12.9)	
				N2	26	>2800	>37			106.5(9.4)	
7	McNicholas *et al*.^98^, 2013	Ireland	1995–1997	L	52	1172(219)	29.9(2.8)	WISC-IV	11	89.7(12.5)	cohort study
				N	48	NA	NA			101.3(11.7)	
8	Cheong *et al*.^39^, 2013	Australia	1991–1992	L	148	897(177)	25.8(1.1)	WASI	18	95.7(15.9)	cohort study
				N	132	3441(457)	39.3(1.3)			107.6(12.8)	
9	Hutchinson *et al*.^99^, 2013	Australia	1997	L	189	833(164)	26.5(2.0)	WISC-IV	8	93.1(16.1)	cohort study
				N	173	3506(1455)	39.3(1.1)			105.6(12.4)	
10	Lundequist *et al*.^100^, 2013	Sweden	1988–1993	L	145	1050(266)	28.1(2.8)	WPPSI-R	5	95.7(16.1)	cohort study
				N	117	3493(453)	39.8(1.2)			102.3(11.0)	
11	Aarnoudse-Moens *et al*.^6^, 2013	Netherlands	1996–2004	L	200	1013(287)	28.1(1.4)	WISC-III	8	93.3(15.8)	cohort study
				N	230	3578(482)	39.9(1.2)			105.0(13.4)	
12	Munck *et al*.^101^, 2012	Finland	2001–2006	L	124	1061(260)	28.7(2.8)	WPPSI-R	5	99.3(17.7)	cohort study
				N	168	3659(454)	40.1(1.2)			111.7(14.5)	
13	Pyhala *et al*.^19^, 2011	Finland	1978–1985	L	103	1140(217)	29.3(2.3)	WAIS-III	25	102.2(15.3)	cohort study
				N	105	3609(489)	40.1(1.2)			110.6(12.0)	
14	Potharst *et al*.^22^, 2011	Netherlands	2002–2004	L	104	1045(254)	28.7(1.6)	WPPSI	5	92(17)	cohort study
				N	95	3436(512)	39.8(1.7)			103(11)	
15	Ni *et al*.^7^, 2011	China	2002–2003	L	37	1158(266)	29.5(2.8)	WISC-IV	6	100.1(10.7)	cohort study
				N	22	3162(404)	38.3(1.5)			103.9(11.1)	
16	Løhaugen *et al*.^102^, 2010	Norway	1986–1988	L	55	1217(233)	29.1(2.5)	WAIS-III	19	88(13)	cohort study
				N	81	3707(433)	39.7(1.2)			101(12)	
17	Soria-Pastor *et al*.^40^, 2009	Spain	1996–1998	L	20	1794	30–34	WISC-IV	9	105.8(13.8)	case-control study
				N	22	3392	40			121.9(15.3)	
18	Aarnoudse-Moens *et al*.^103^, 2009	Netherlands	1998–2000	L	50	1042(31)	28.0(1.4)	WPPSI-R	6	92.5(17.5)	case-control study
				N	50	3579(510)	39.7(1.3)			109.0(19.2)	
19	Woodward *et al*.^28^, 2009	New Zealand	1998–2000	L1	43	807(233)	<28	WPPSI-R	4	93.86(17.57)	cohort study
				L2	62	1237(223)	28–33			95.65(13.88)	
				N	107	3574(409)	38–41			104.70(13.45)	
20	Mu *et al*.^104^, 2008	China	1995–1997	L	130	1165(238)	29.5(2.7)	WISC-III	8	93.14(16.33)	case-control study
				N	59	3312(379)	39.3(1.1)			111.05(14.81)	
21	Gaddlin *et al*.^34^, 2008	Sweden	1987–1988	L1	59	1214(212)	30.7(2.4)	WISC-III	15	84.9(17.5)	cohort study
				N1	57	3637(524)	40.2(1.3)			97.1(13.3)	
				L2	31	1213(191)	32.0(2.5)			84.1(19.9)	
				N2	28	3477(440)	39.9(1.0)			85.7(14.7)	
22	Allin *et al*.^18^, 2008	UK	1982–1984	L	94	<2500	<33	WASI	19	96.6(13.7)	cohort study
				N	44	NA	38–42			105.3(12.8)	
23	Saavalainen *et al*.^105^, 2007	Finland	1984–1986	L	35	1440(440)	30(2)	WISC-R	9	96.3(11.3)	cohort study
				N	31	3427(410)	40(1.3)			100.3(10.6)	
24	Nosarti *et al*.^106^, 2007	UK	1979–1982	L	61	1296(295)	29.5(1.8)	WASI	22	105.14(11.99)	cohort study
				N	64	>2500	37–42			111.75(10.56)	
25	Narberhaus *et al*.^32^, 2007	Spain	1983–1994	L1	9	899	26.4	WISC-R or WAIS-III	14	91.4(14.4)	case-control study
				L2	19	1140	29			100.5(16.2)	
				L3	25	1534	31.7			103.2(15.7)	
				L4	11	2445	34.6			112.7(13.8)	
				N	53	3416	39.6			113.6(11.5)	
26	Hoff *et al*.^107^, 2006	Denmark	1994–1995	L	191	922(167)	27.5(1.8)	WPPSI-R	5	96.4(14.1)	cohort study
				N	76	3530(518)	40.1(1.2)			107.3(11.4)	
27	Martinez-Cruz *et al*.^27^, 2006	Mexico	1997	L1	25	875(107)	31.4(1.7)	Stanford-Binet	6	95.3(11.3)	case-control study
				L2	52	1297(130)	32.5(1.2)			103.1(14.4)	
				L3	66	1940(247)	33.6(1.8)			105.1(12.3)	
				N	41	3239(410)	39.6(1.8)			106.8(11.7)	
28	Hack *et al*.^24^, 2005	USA	1992–1995	L	219	810(124)	26.4(2)	KABC	8	87.8(18)	cohort study
				N	176	3300(513)	≥37			99.8(15)	
29	Lefebvre *et al*.^21^, 2005	France	1976–1981	L	69	912(79)	28.5(2.4)	WAIS	18	94(12)	cohort study
				N	44	3419(418)	39.8(1.1)			108(14)	
30	Marlow *et al*.^11^, 2005	UK	1995	L	241	<2500	<26	K-ABC	6	82.1(19.2)	cohort study
				N	160	NA	>37			105.7(11.8)	
31	Kilbride *et al*.^108^, 2004	USA	1983–1990	L	25	702(76)	26.0(1.6)	Stanford-Binet	4	85(12)	cohort study
				N	25	3215(509)	38.8(1.5)			95(11)	
32	Short *et al*.^109^, 2003	USA	1989–1991	L	75	1256(176)	30(2)	WISC-III	8	91.7(16)	cohort study
				N	99	3451(547)	40(1)			101.9(15)	
33	Cooke *et al*.^110^, 2003	UK	1991–1992	L	268	1467	29.8	WISC-III	7	89.4(14.2)	cohort study
				N	198	NA	NA			100.5(13.7)	
34	Grunau *et al*.^111^, 2002	Canada	1982–1987	L	74	718(480–800)	26.0(23–33)	WISC-R	9	99.3(10.9)	cohort study
				N	30	3540(2948–4706)	40.0(28–40)			117.3(13.0)	
35	Magill-Evans *et al*.^112^, 2002	Canada	NA	L	20	2104	<37	WISC-III	10	98(14.9)	cohort study
				N	23	3515	NA			101.5(11.9)	
36	Breslau *et al*.^17^, 2001	USA	1983–1985	L1	231	<2500	NA	WISC-R	11	88.1(14.7)	cohort study
				N1	143	≥2500				94.1(13.6)	
				L2	180	<2500				107.8(14.8)	
				N2	163	≥2500				112.8(14.3)	
37	Rickards *et al*.^113^, 2001	Australia	1980–1982	L	120	1167(215)	29.3(2.0)	WISC-III	14	96.2(15.5)	cohort study
				N	41	3417(432)	39.9(1.0)			105.0(13.3)	
38	Nadeau *et al*.^114^, 2001	Canada	1987–1990	L	61	1024(204)	27.4(1.1)	MIQS	5	100.3(19.1)	cohort study
				N	44	3453(498)	39.8(1.6)			112.8(16.2)	
39	Taylor *et al*.^115^, 2000	USA	1982–1986	L1	60	665(68)	25.7(1.8)	KABC	11	83.49(19.7)	cohort study
				L2	55	1173(217)	29.4(2.4)			96.81(14.4)	
				N	49	3300(660)	40			106.24(14.3)	
40	Tandon *et al*.^33^, 2000	India	1985–1995	L1	27	1810(248)	36.2(2.9)	Stanford-Binet	8	105.6(13.4)	cohort study
				N1	28	2850(363)	39.2(1.2)			116(11.6)	
				L2	32	1740(195)	36(2.5)			99.6(11.8)	
				N2	29	2850(331)	39.8(1.3)		11	110.6(7.3)	
41	Saigal *et al*.^116^, 2000	Canada	1977–1982	L	150	833(126)	27(2.4)	WISC-R	14	89(19)	cohort study
				N	124	3395(483)	40			102(13)	
42	Hughes *et al*.^117^, 1999	USA	1979–1981	L1	95	964(208)	28.5(2.1)	WISC-R	9	86.16(17.67)	cohort study
				L2	311	1157(272)	30.6(2.3)			95.56(17.63)	
				N	188	2776(707)	39.4(1.7)			99.79(16.51)	
43	Stjernqvist *et al*.^118^, 1999	Sweden	1985–1986	L	61	1042(242)	27.1(1.03)	WISC-III-R	10	89.8(15.1)	cohort study
				N	61	3648(533)	40.1(1.43)			106.5(15.0)	
44	Botting *et al*.^119^, 1998	UK	1980–1983	L	138	<1500	NA	WISC-III	12	89.7(17.2)	cohort study
				N	163	>2500	>37			97.8(17.4)	
45	Whitfield *et al*.^120^, 1997	Canada	1974–1985	L	90	731(520–800)	26(23–28)	WISC or Stanford-Binet	9	98.7(12.6)	cohort study
				N	50	3488(2614–4706)	40(38–42)			111.6(13.1)	
46	Rose *et al*.^121^, 1996	USA	1979–1981	L	50	1154(233)	31.2(1.8)	WISC-R	11	89.6(11.3)	cohort study
				N	40	NA	NA			98.9(11.9)	
47	Sommerfelt *et al*.^46^, 1995	Norway	1986–1988	L	144	1555(368)	32(3)	WPPSI-R	5	97(14)	cohort study
				N	163	>3000	40			104(14)	
48	Levy-Shiff *et al*.^61^, 1994	Israel	NA	L	90	1190(209)	29(2.3)	WISC-R	7	105.1(10.5)	cohort study
			NA	N	90	3225(334)	39(1.2)			114.4(9.8)	
49	Sommerfelt *et al*.^14^, 1993	Norway	1981–1982	L	29	1251(166)	31.1(2.6)	WISC-R	8	93.2(16)	cohort study
				N	29	3650(490)	40(0)			104.2(14)	
50	Hack *et al*.^122^, 1992	USA	1977–1979	L	249	1176	29.2	WISC-R	8	95.7(18)	cohort study
				N	363	>2500	>37			100.6(17.6)	
51	Teplin *et al*.^123^, 1991	USA	1980	L	28	905(86)	28(1.5)	KABC	6	86.3(13.6)	cohort study
				N	26	NA	>37			98.7(14.3)	
52	Smith *et al*.^124^, 1990	UK	1981	L	43	1306(164)	> 28	MIQS	5	88.56(16.94)	cohort study
				N	43	3342(429)	>37			101(13.04)	
53	McDonald *et al*.^125^, 1989	USA	NA	L	16	1776(510)	31.4(3)	WPPSI	5	113(21)	cohort study
				N	18	3359(481)	40			124(13)	
54	Klein *et al*.^126^, 1989	USA	1976	L	65	1190(197)	30(2)	WISC-R	9	92(14)	cohort study
				N	65	>3000	>37			98(16)	
55	Portnoy *et al*.^127^, 1988	UK	1980–1981	L	15	909	NA	MIQS	5	93(20)	cohort study
				N	15	>2500	38–42			103(13)	
56	Lloyd BW *et al*.^128^, 1988	UK	1978–79	L	44	1302	26–37	BAS	7	93.1(15)	cohort study
				N	44		40			100.4(12.9)	
57	Kitchen WH *et al*.^129^, 1980	Australia	1966–1970	L1	10	<1000	NA	WISC-R	8	79.4(15.7)	cohort study
				L2	143	<1501	NA			89.4(15.7)	
				N	43	>2500	NA			98.8(15.7)	

LBW: low birth weight; NBW: normal birth weight; NA: not available; L: LBW; N: NBW; WISC-III: Wechsler Intelligence Scale for Children, Third Edition; WISC-R: Wechsler Intelligence Scale for Children, Revised; WISC-IV: Wechsler Intelligence Scale for Children, Fourth Edition; WPPSI: Wechsler Preschool and Primary Scales of Intelligence Test; WPPSI-R: Wechsler Preschool and Primary scale of intelligence, Revised; WASI: Wechsler Abbreviated Scales of Intelligence; WAIS-III: Wechsler Adult Intelligence Scale; KABC, Kaufman Assessment Battery for Children; K-Bit: Kauf-man Brief Intelligence Test, Italian version; MIQS: McCarthy IQ Scale; BAS: British Abilities Scale.

### Overall analysis

**Figure 2:**
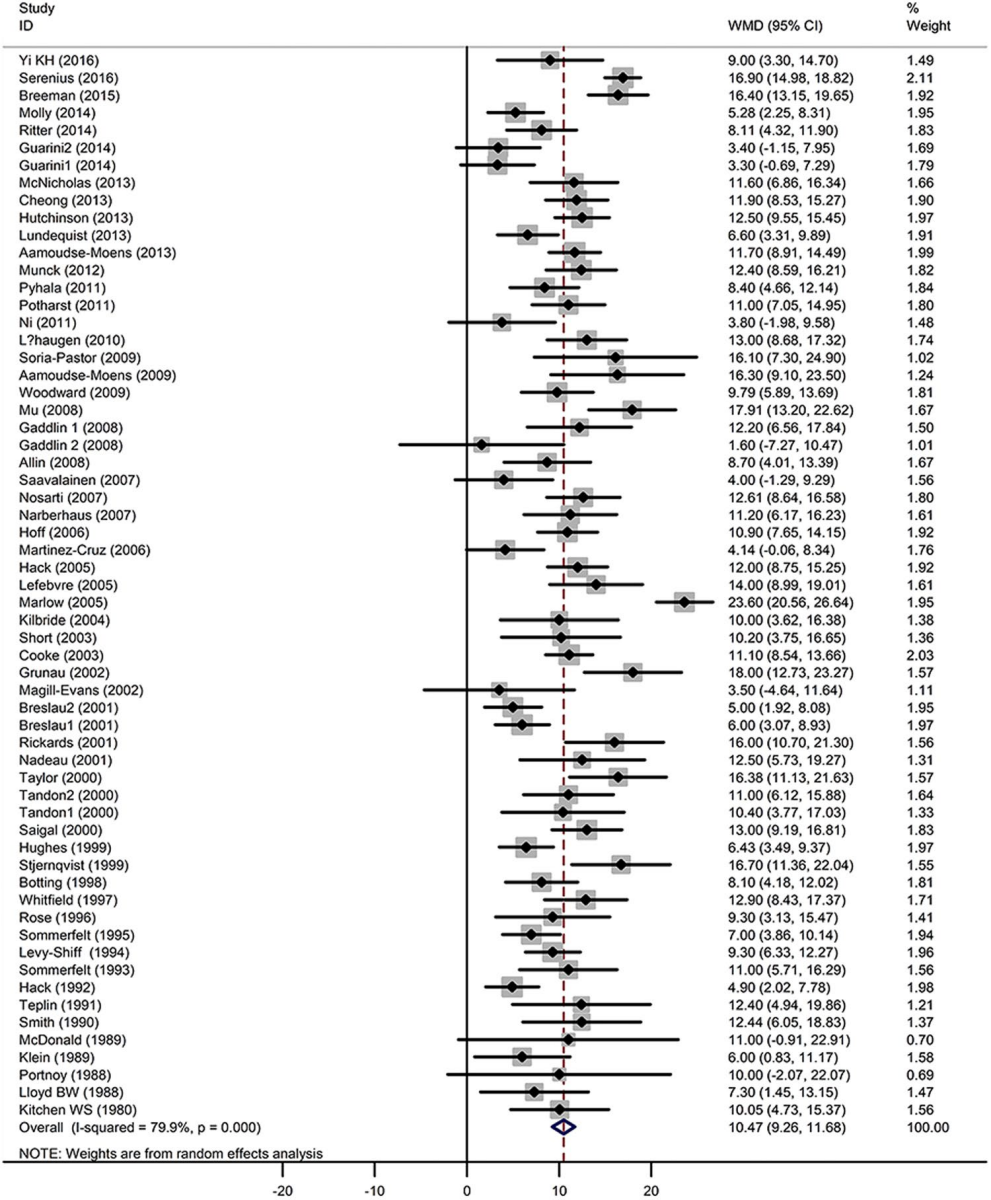
Random-effect analysis of the association between low birth weight and IQs. WMD: weight mean difference; CI:confidence interval.

All studies revealed that the LBW individuals had lower IQs compared with the NBW group. The pooled WMD was 10 (Z = 17.12, *P* < 0.001), with a 95% CI of 9.26–11.68, which means that the LBW individuals’ IQs were significantly lower than those of the NBW controls (Fig. 2). Between-study heterogeneity was detected [Q = 298.79 (*P* < 0.001) and I^2^ = 79.9% (*P* < *0.001*)]. The mean IQs of the ELBW, VLBW, LBW and NBW individuals were 91, 94, 99 and 104, respectively. A gradient relationship was observed between birth weight and IQ.

### Sensitivity analysis and publication bias

**Figure 3:**
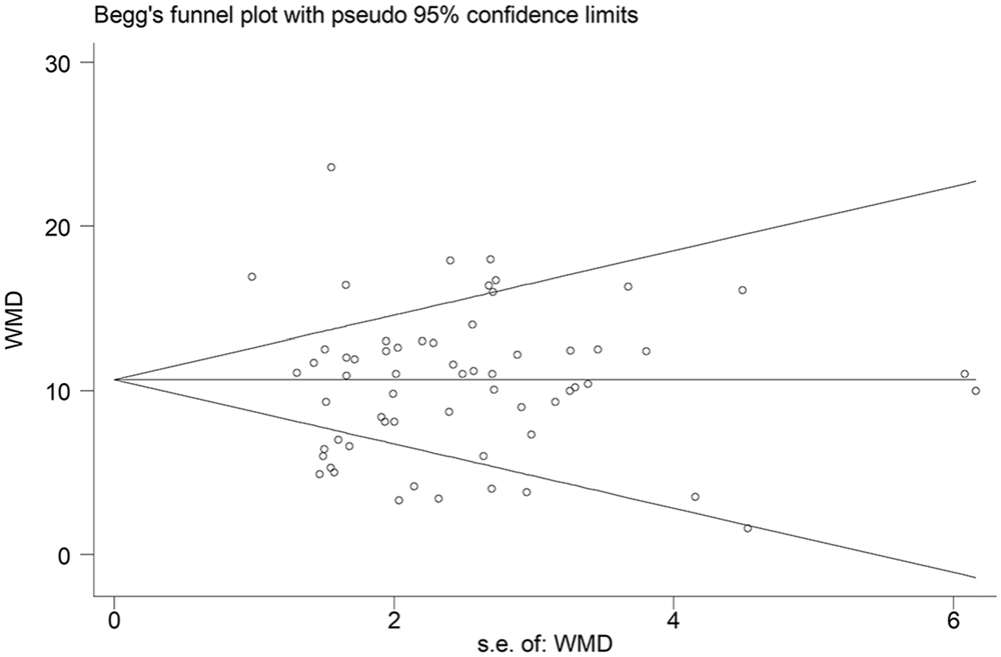
Begg’s funnel plot of individual studies included in the analysis according to random-effect WMD estimates.

After excluding one study at a time, the sensitivity analysis confirmed the significant association between LBW and IQs (with 95% CI ranging from 0.68 to 0.76) (Figure S1). No publication bias was detected (Begg’s test: *P* = 0.49 and Egger’s test: *P* = 0.50). Figure 3 shows a basic funnel plot depicting potential bias.

### Sources of heterogeneity

**Figure 4:**
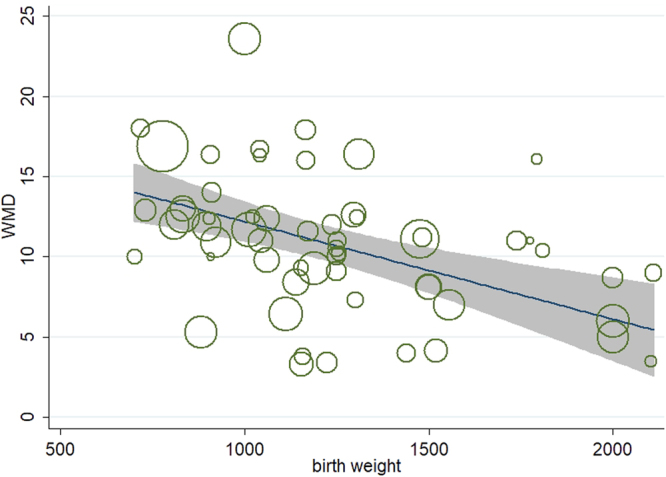
Meta-regression of birth weight on IQs difference between NBW and LBW individuals.

We used meta-regression models to probe the source of heterogeneity. The variables included sample size, birth year, age of assessment, and the birth weight of LBW individuals. The model showed that the birth weight of the LBW participants was associated with an IQ difference between NBW and LBW individuals (coefficient = −0.005, adjusted R^2^ = 13.22%, *P *=0.003). Other variables did not reach the significance level (Table S1). Low birth weight contributed to 30.5% of the heterogeneity after further analysis, with T^2^ reduced from 17.10 to 11.88. Figure 4 shows the meta-regression model of the effect of low birth weight on IQ. The results from the Galbraith plot (Figure S2) indicated that two populations (Serenius^10^, Marlow^11^) with the highest WMD may be the main cause of high heterogeneity. After excluding these two studies, the adjusted pooled WMD was 10 (95% CI 9.02–11.03, I^2^ = 67.4%, *P* < 0.01). Approximately 15.6% of the heterogeneity was attributable to these two studies.

### Subgroup analysis

We performed subgroup analysis to examine whether a gradient relationship existed between different LBW levels and IQs. As shown in Table 2, the WMD was 7 (95% CI 4.76–8.89), 10 (95% CI 8.43–11.28), and 14 (95% CI 11.71–16.20) for MLBW, VLBW and ELBW, respectively (Figures S3–S5). To identify age-related changes in IQs between LBW and NBW individuals, all studies were divided into three groups, i.e., under 10 years, 10–18 years, and over 18 years. The WMD was 11 (95% CI 8.87–12.30), 10 (95% CI 7.88–11.75), and 11 (95% CI 8.42–11.68), respectively. Thus, the discrepancy was stable regardless of age (Table 2; Figure S6).

**Table 2:** Subgroups analysis based on birth weight and age at assessment.

	No. of study group	WMD	95% CI	Z test *P* value	*I* ^2^ (%)	Q test P value	Egg’s test *P* value
birth weight							
<1000	20	13.95	11.71–16.20	<0.001	81.7	<0.001	0.739
1000–1499	35	9.85	8.43–11.28	<0.001	70.6	<0.001	0.764
1500–2499	11	6.83	4.76–8.89	<0.001	43.7	0.06	0.382
age							
<10	36	10.58	8.87–12.30	<0.001	83.5	<0.001	0.278
10~18	17	9.82	7.88–11.75	<0.001	66.4	<0.001	0.151
≥18	8	11.28	8.42–11.68	<0.001	76.8	<0.001	0.588
social determinants							
matched	39	9.90	8.42–11.39	<0.001	76.8	<0.001	0.172
non-matched	22	11.42	9.31–13.53	<0.001	84.4	<0.001	0.409

Another subgroup meta-analysis was based on social determinants of health. The LBW and NBW groups were matched by social determinants of health in 39 individual studies, whereas other studies had different social determinant distributions for the two groups. The results showed that the WMDs between NBW and LBW individuals were 10 (95% CI 8.42–11.39) and 11 (95% CI 9.31–13.53) for social determinants between matched groups and non-matched groups (Figure S7), respectively. Therefore, approximately 13% of the IQ discrepancy was due to social determinants of health.

## Discussion

Our study supported the evidence that individuals’ low birth weight had a negative association with IQ^4,29^. The lower birth weight categories had lower IQs on average. The average IQs of ELBW individuals were the lowest, followed by VLBW individuals and those with MLBW. Specifically, low birth weight individuals had approximately 10–11 points lower IQs than NBW individuals from childhood to adulthood (4–26 in age). There was a gradient relationship between low birth weight and the discrepancy in IQs between LBW and NBW individuals, with the WMDs from large to small being 14 (ELBW), 10 (VLBW), and 7 (MLBW). In addition, social determinants of health were associated with individuals’ IQs, which explained approximately 13% of the IQ difference between LBW and NBW individuals.

The gradient relationship obviously depicted the IQ gap between individuals with different levels of LBW and those with NBW. The M LBW infants were closer to preterm (<37 weeks)^29^, while the VLBW and ELBW infants tended to be less than 32 weeks in gestational age^3^. Because of the high degree of immaturity of respiratory organs and the nervous system, they were susceptible to bronchopulmonary dysplasia^35^, neonatal brain injury (cerebral palsy, periventricular leukomalacia, hydrocephalus, hypoxic-ischaemic encephalopathy)^9,35–37^, and other medical complications, which may result in cognitive impairment. Additionally, children born with low birth weight had less connected and less complex brain networks^38^, smaller brain volumes^39–41^, and less cortical surface area^42^ compared with NBW children. The different degree of neonatal immaturity in LBW infants is considered to be associated with cognitive outcomes^34,43^.

We also found a stable difference in IQs between LBW individuals and NBW individuals. The discrepancy was approximately 10–11 points regardless of the age of assessment. This finding was inconsistent with previous reports that showed that the discrepancy would decrease over time^29^. Some LBW individuals may have cognitive catch-up growth^44^, but it is not a universal rule among those with LBW. A long-term follow-up study on a population sample aged from 5 months to 26 years showed that the IQs were more stable in very preterm (VP)/VLBW individuals than in term-born individuals across all time points^45^. However, this conclusion was based on the entire LBW group and may not be applicable to a single individual.

Social determinants of health, such as social class, parental/maternal education and occupation, marital status, etc., are known to contribute to suboptimal cognitive development of LBW children. Previous studies have indicated that LBW continues to be associated with cognitive disadvantage at each SES level^21^ and that the risk of impaired cognitive development increases with decreasing SES^46^. A study by Sommerfelt *et al*. reported that 23% of the variance in child’s IQ at age 5 could be attributed to parental and family variables in Norway. Our results showed that social determinants of health explained approximately 13% of the lower IQ values. Because of the diversity of social determinants in different societies and the variations in study design, the common practice of simply matching social determinants of health (social class, occupation, parental/maternal education) may result in an underestimation of cognitive impairment caused by social determinants of health or other similar risk factors.

Intelligence is a product of genetic and environmental variables^47^.Genetic variation is the main cause of individual differences in IQ^48^. Previous studies have reported that the “heritability” (*h* ^2^) for IQ ranges from 20% in infancy, to 40–50% by late adolescence and to 60–80% in adulthood^49^. Environmental factors, such as perinatal factors^50^, schooling, family environment, nutrition and so on^49^ also contribute to individuals’ IQs. The aetiology of LBW individuals’ lower IQs is complex and unclear. Various adversities occur among LBW infants, such as preterm birth, the stress of intensive care and more frequent morbidities, which may also affect individuals’ IQs. It may be that low birth weight is an event along this causal pathway. However, two cohort studies from Denmark^51^ and Estonia^52^ demonstrated the associations between birth weight and IQs, and the associations remained significant after controlling for a wide range of confounders. These correlations were modest, ranging from 0.05 to 0.13^52,53^.

As poor cognitive outcomes may be related to lower school achievements^12,54^, inferior SES^5^, an unhealthy lifestyle^47,55,5^, and even some chronic diseases^56^, improving the cognitive outcome of LBW infants is essential and urgent. Previous evidence showed that the LBW individuals can benefit from early interventions for cognitive outcomes^3,9^. Some randomized controlled trials, such as the Newborn Individualized Developmental Care and Assessment Program (NIDCAP)^57^ and a sensitizing parental intervention programme^58^, showed that breastfeeding^59^ and kangaroo care had beneficial effects on LBW infants’ cognitive outcomes. It is recommended to assess the cognitive ability of LBW individuals first in order to determine the need for interventions. Periodic cognitive assessment of LBW children can evaluate the intervention’s effectiveness, thus providing more accurate interventions for each individual^60^. The cognitive benefits from early intervention may persist into preschool age or adolescence^58^. Therefore, long-term interventions may play a role in the long run. Although there were few long-term intervention programmes reported, it is necessary for child care centres and parents to offer long-term neuropsychological rehabilitation to LBW individuals even if they do not suffer from severe cognitive disabilities.

### Strengths and limitations

#### Strengths

Compared with previous meta-analyses of LBW/preterm individuals’ IQs, we included more eligible and recent individual studies, with a total of 12,137 participants and without a duplicated study population. We conducted subgroup analyses to show the gradient in the IQ gap between individuals with different levels of LBW and those with NBW, as well as the stability of the difference in IQs between LBW and NBW individuals. Although the selected studies used different cognitive tests to measure individuals’ IQs, each test/scale had similar normative data (mean = 100; SD = 15), which made the results from different studies comparable.

#### Limitations

We tried to include all relevant studies, but some studies may be missed in this meta-analysis due to our search strategies or incomplete databases. Additionally, grey literature publications were not included. However, the large sample size of this study made the results more stable and credible.

According to individual studies, parental/maternal education was either a variable of socio-economic status or an independent social determinant. Three individual studies only matched by parental/maternal education were also included in the social determinant-matched group. Since there is not a perfect fit between education and socio-economic status, residual confounding may exist in the subgroup analysis based on social determinants of health.

IQ is a complex trait that is influenced by genetic and environmental factors, such as parental IQs^17,45^, medical complications^61^, early home environment, schooling, and so on^36,60,62^. We didn’t take this residual confounding into account. These factors may also contribute to the heterogeneity. The association between these factors and LBW IQs will be explored in a further study.

## Methods

### Literature and search strategy

We searched the PubMed and the Embase databases for full-text articles in English published between January 1980 and November 2016. The following terms were used to perform the literature search: “low birth weight” or “preterm” or “premature”, and “intelligent quotient” or “IQ” or “cogni*” or “neuro*” or “mental” or “psycho*” or “outcome”.

### Inclusion criteria

Each study should meet all of the inclusion criteria.

1. Participants with LBW (< 2500 g) were compared with those with NBW (≥ 2500 g).
2. The individuals’ ages were ≥ 4 years.
3. Full-scale IQ was measured by a standardized and global scale with the mean and standard deviation of the IQs listed.
4. Full-text articles were available from the two databases.

We excluded reviews, studies of the non-LBW group, and those without NBW individuals as a control group. If more than one study was based on the same cohort, only the study with the larger sample was included in the meta-analysis. When the study had two or more LBW groups, we calculated the weighted mean and deviation to represent the LBW individuals’ IQs in the meta-analysis (Figure S8). For the subgroup analysis, we used the raw data from each study.

### Data extraction

The following information was extracted from each study:

(1) first author’s name; (2) year of publication; (3) country of origin; (4) birth year of the participants; (5) size of study population; (6) birth weight; (7) gestational age; (8) measurement tools; (9) age at assessment; and (10) mean and standard deviation of the IQs.

### Statistical analysis

A random-effects meta-analysis was performed using the WMD in IQs between LBW and NBW individuals. The significance of the WMD was determined using a Z test (*P* < 0.05 was considered statistically significant). To assess the heterogeneity, we consulted the Cochrane Q test and I^2^ statistics. Publication bias was assessed by Begg’s test and Egger’s test. We also used a funnel plot to depict the potential publication bias. We constructed meta-regression models and a Galbraith radial plot to probe the source of heterogeneity.

The subgroup analysis was conducted based on birth weight, age at assessment and social determinants of health. In the first subgroup analysis, we divided the studies into three subgroups according to the LBW participants’ birth weight, i.e., moderately low birth weight (MLBW, 1500–2499 g), very low birth weight (VLBW, 1000–1499 g) and extremely low birth weight (ELBW, <1000 g). Then, we grouped individual studies into three groups by the subjects’ age at assessment (under 10 years, 10 to 18 years, 18 years or older) in the second subgroup analysis. Because social determinants of health are associated with individuals’ IQs, we compared the social determinant-matched group with the social determinant-unmatched group to evaluate how much of the lower IQ values were due to social determinants of health. All analyses were conducted using STATA version 11 (Stata Corp LP, College Station, Texas, USA).

## Conclusion

Individuals with LBW had lower IQs compared to those with NBW, and the discrepancy was approximately 10–11 points from childhood to adulthood (4–26 in age). We also demonstrated a gradient relationship between different levels of LBW and IQs. The social determinants of health explained approximately 13% of the IQ difference. These findings contribute to our understanding of the association between LBW and IQs. Our results will help physicians and parents to pay more attention to regular cognitive assessment and early intervention, as well as to long-term neuropsychological rehabilitation for LBW infants.

## Electronic supplementary material

supplementary information
