# Supplementary material for: A gradient relationship between low birth weight and IQ: A meta-analysis
Source: Sci Rep. 2017 Dec 21;7:18035. doi: 10.1038/s41598-017-18234-9 (PMC5740123; doi:10.1038/s41598-017-18234-9)
Supplement: Supplementary file 1 — supplementary information [file 41598_2017_18234_MOESM1_ESM.pdf]

A gradient relationship between low birth weight and IQ: A meta-analysis

Huaiting Gu [1, 2 #], Lixia Wang [3 #], Lingfei Liu [1], Xiu Luo [1], Jia Wang [1], Fang Hou [1], Pauline Denis Nkomola [1], Jing Li [2], Genyi Liu [2], Heng Meng [1], Jiajia Zhang [4], Ranran Song [1 *]

[#] G. H. and W. L. contributed equally to the work.

1. Department of Maternal and Child Health, and MOE (Ministry of Education) Key Laboratory of Environment and Health, School of Public Health, Tongji Medical College, Huazhong University of Science and Technology, Wuhan, 430030, China.
2. School of Public Health, Jining Medical College, Jining, 272067, China.
3. Department of Radiology, Union Hospital, Tongji Medical College, Huazhong University of Science and Technology, Wuhan, 430030, China.
4. Department of Epidemiology and Biostatistics, Arnold School of Public Health, University of South Carolina, Columbia, 29208, USA.

*Correspondences: Ranran Song, PhD, Department of Maternal and Child Health, and MOE (Ministry of Education) Key Laboratory of Environment and Health, School of Public Health, Tongji Medical College, Huazhong University of Science and Technology, Wuhan, 430030, China.

E-mail: songranran@hust.edu.cn; Tel: +86-27-83657509; Fax: +86-27-83657509.

Table S1 Results of meta-regression

Covariate	coefficient	95% CI	t-value	P-value
sample size of LBW	0.004	-0.019, 0.028	0.37	0.709
sample size of NBW	-0.004	-0.030, 0.021	-0.36	0.723
age at assessment	0.1098	-0.003, 0.961	0.94	0.352
birth weight	-0.005	-0.009, -0.002	-3.09	**0.003**
birth year	0.040	-0.106, 0.186	0.55	0.586

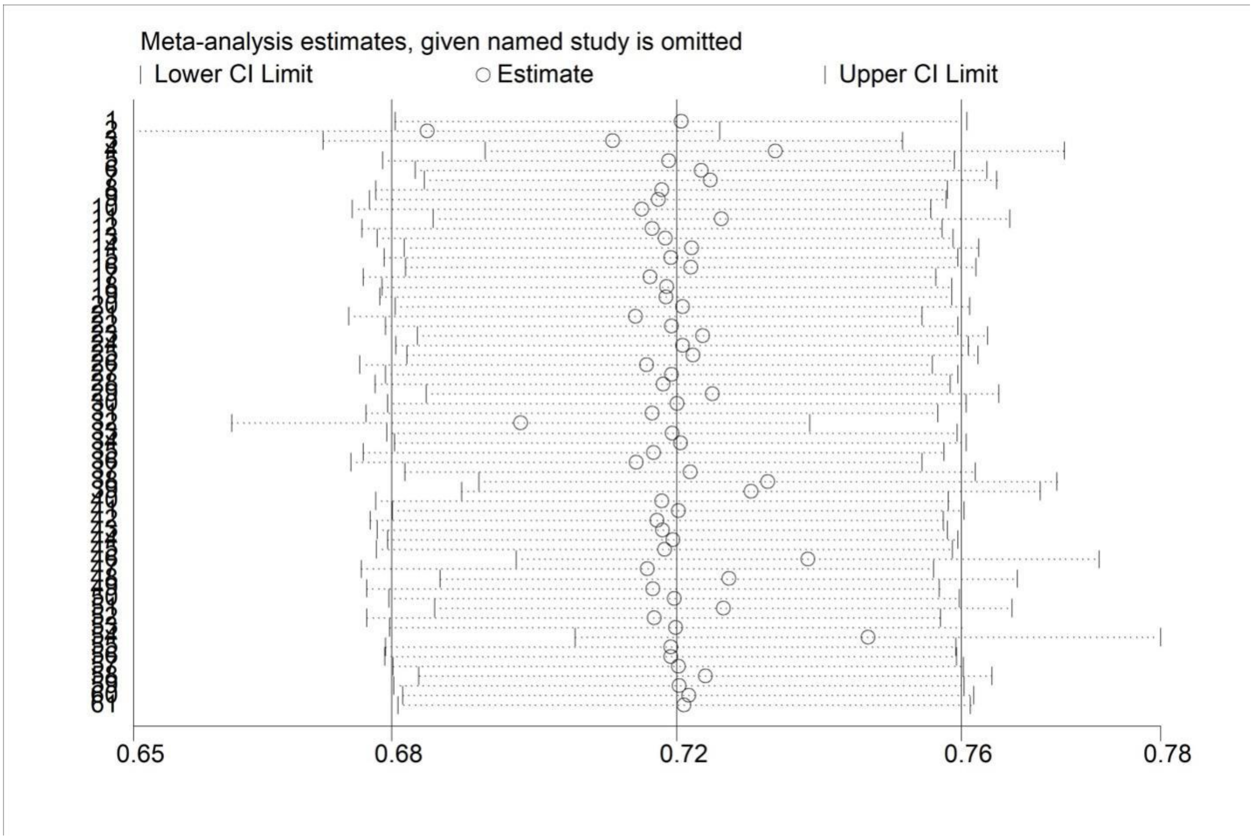

Figure S1 Sensitivity analysis for individual studies on the summary effect.

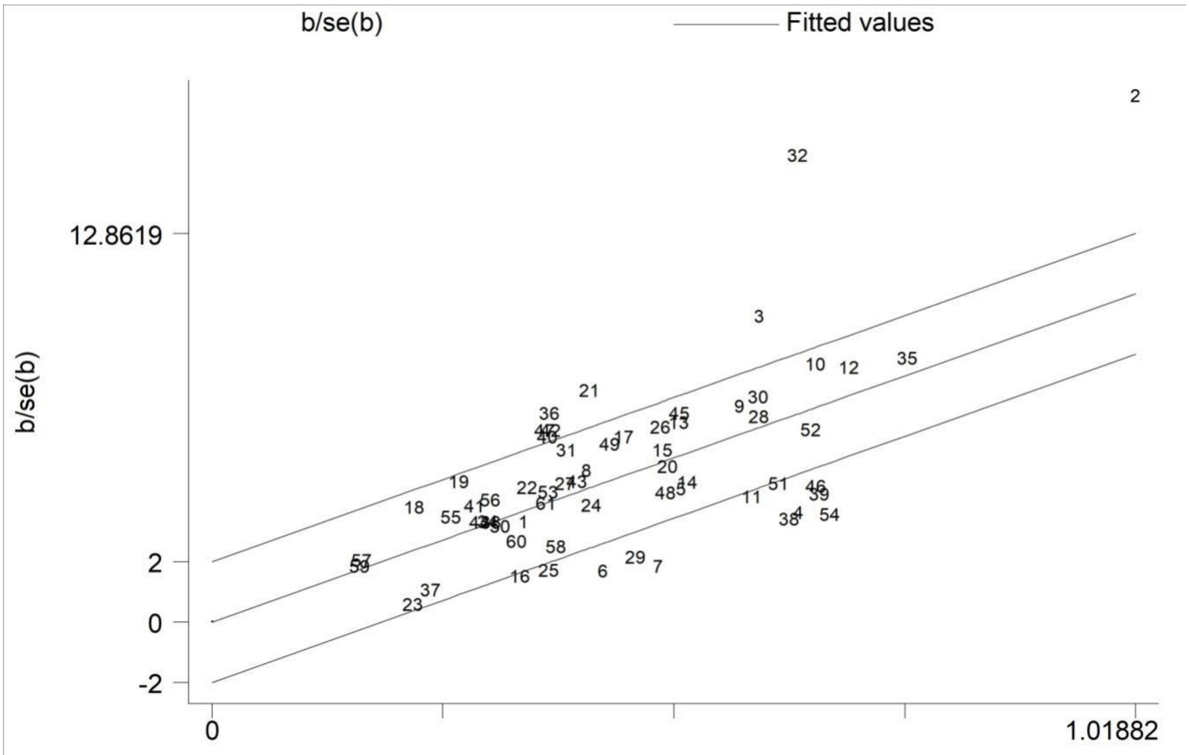

Figure S2 Galbraith plot

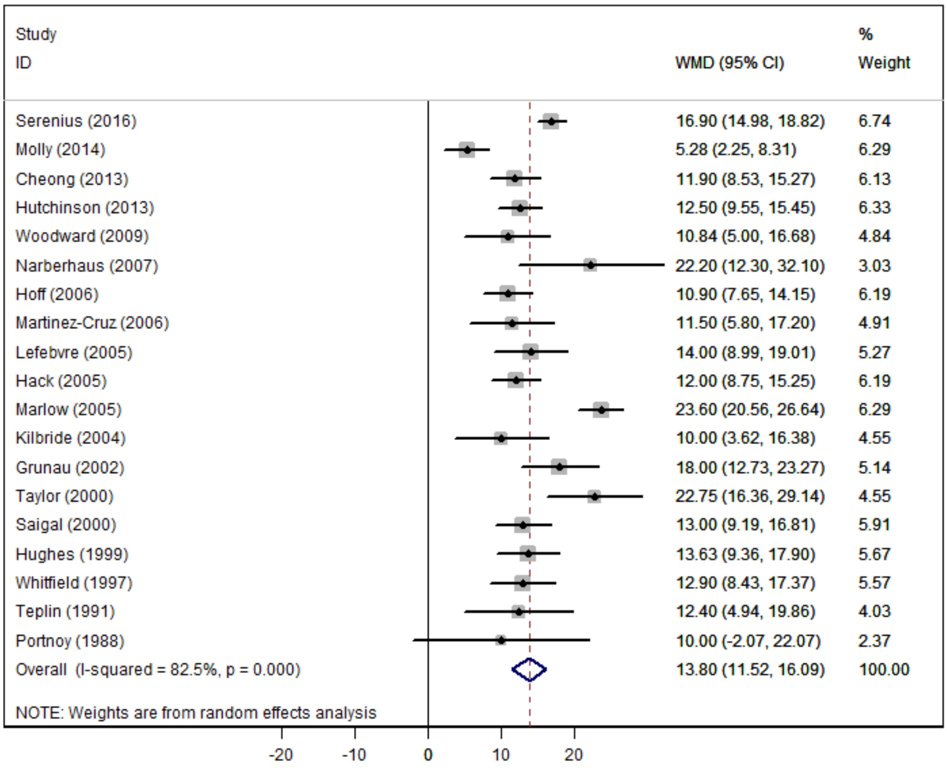

Figure S3 Forest plot of pooled WMD for ELBW (<1000g)

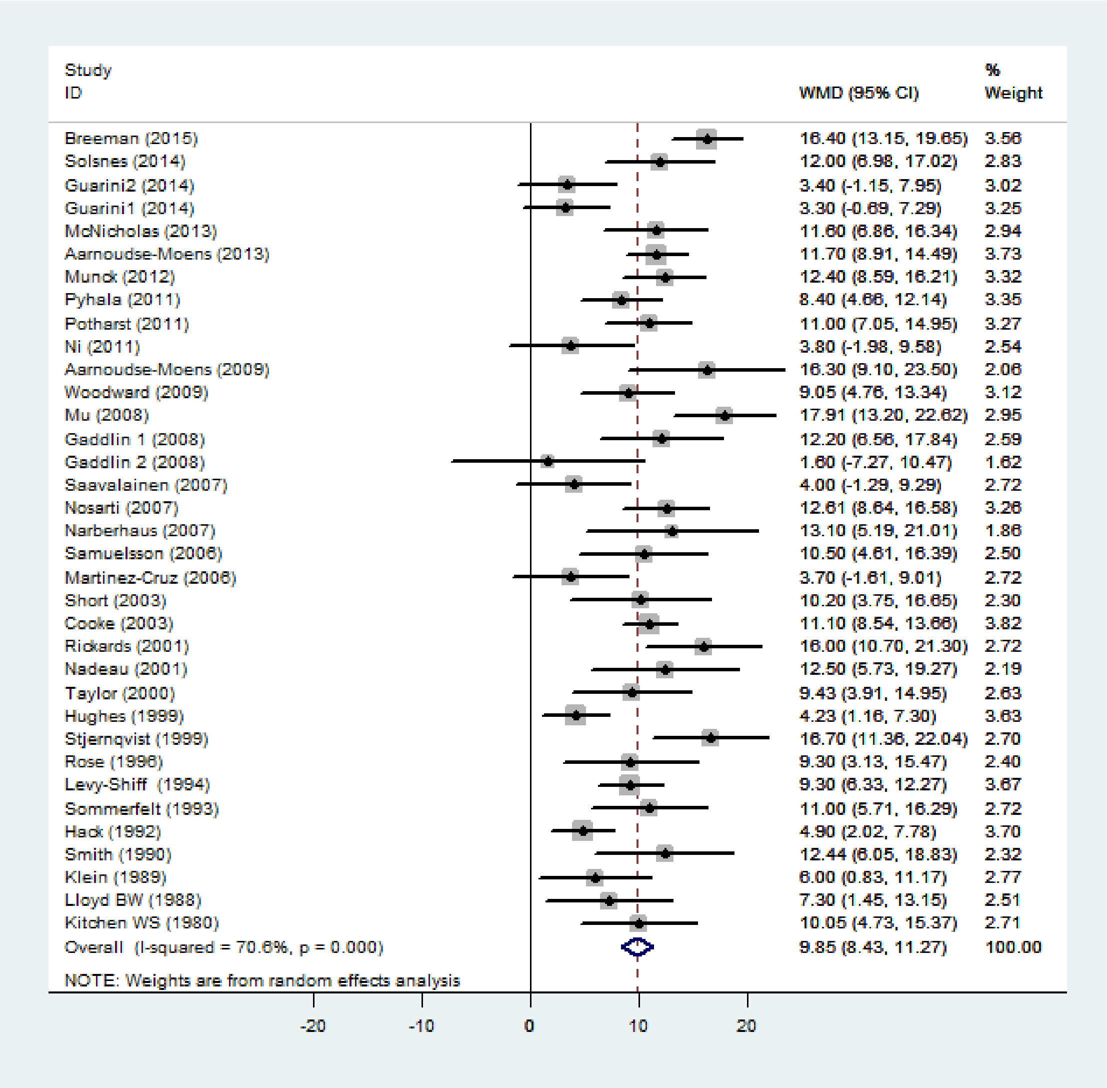

Figure S4 Forest plot of pooled WMD for VLBW (1000---1499g)

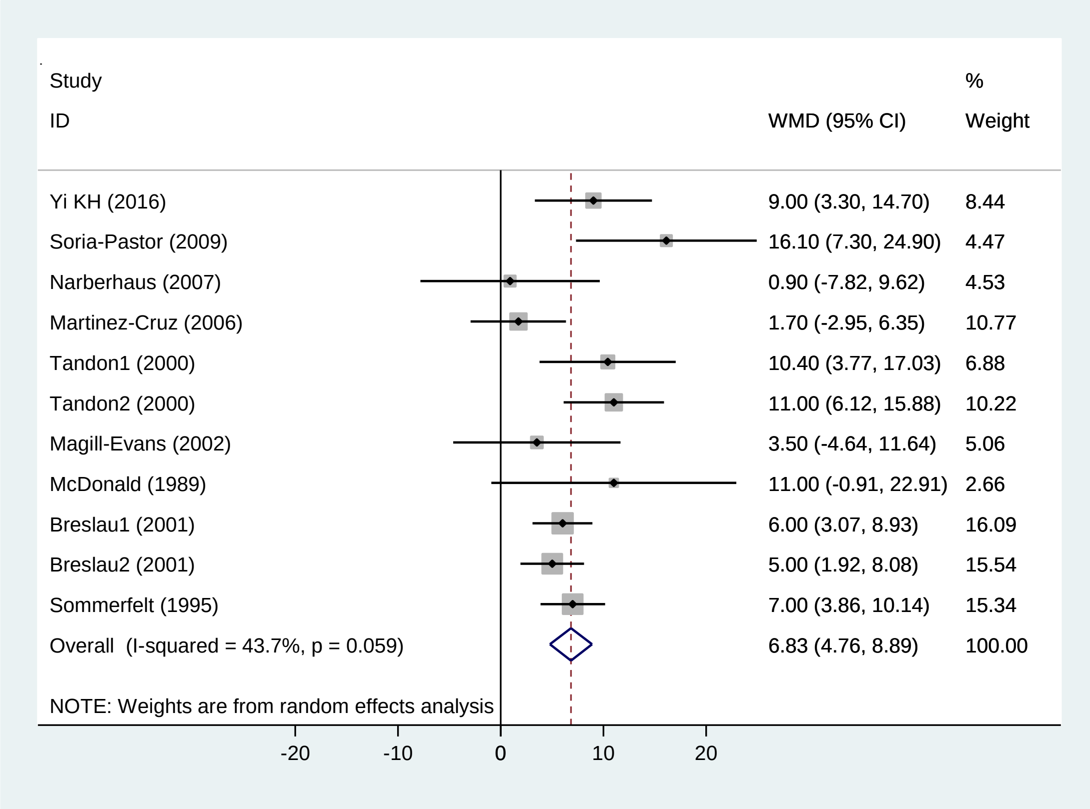

Figure S5 Forest plot of pooled WMD for MLBW (1500---2499g)

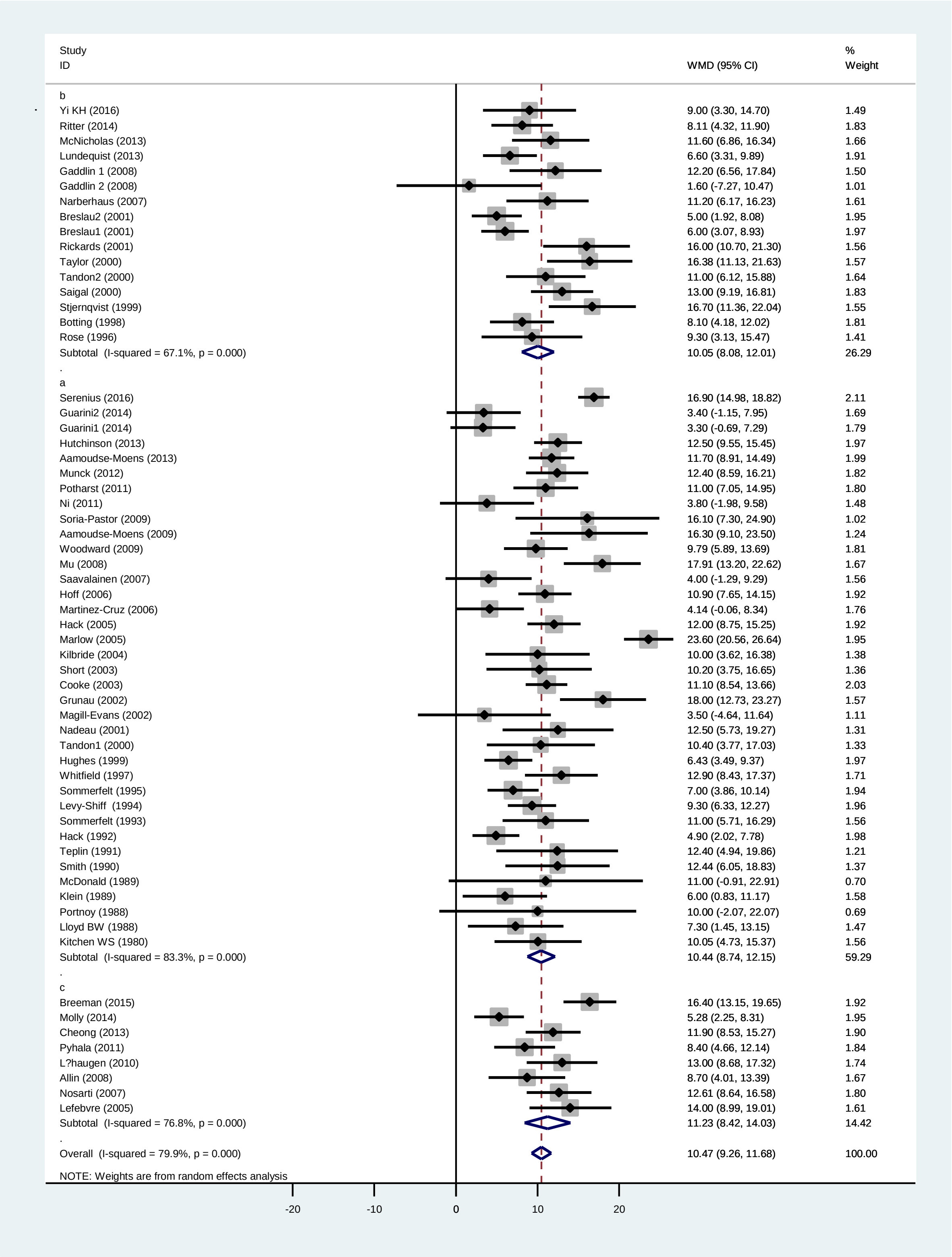

Figure S6 Forest plot of pooled WMD by age groups

a : < 10 years old; b: 10 to18 years old; c: ≥18 years old

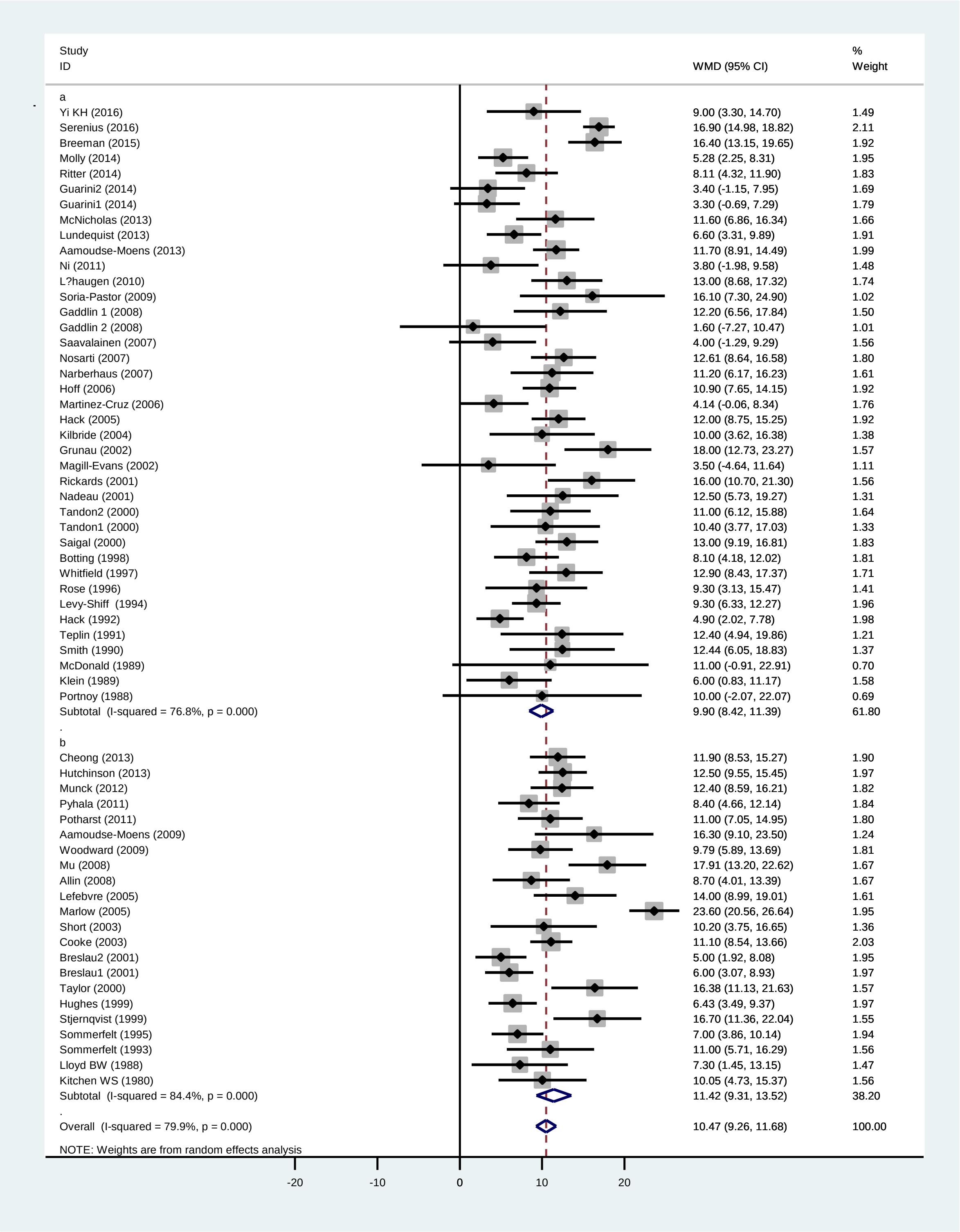

Figure S7 Forest plot of pooled WMD for the social determinants of health (SDOH) matched group and SDOH non-matched group.

a : SDOH matched group;

b : SDOH non-matched group.

	group 1	group 2	group (1+2)
Sample Size	N_1	N_2	$N_1 + N_2$
Mean	M_1	M_2	$\frac{N_1M_1 + N_2M_2}{N_1 + N_2}$
Standard Deviation	SD_1	SD_2	$\sqrt{\frac{(N_1 - 1)SD_1^2 + (N_2 - 1)SD_2^2 + \frac{N_1N_2}{N_1 + N_2}(M_1^2 + M_2^2 - 2M_1M_2)}{N_1 + N_2 - 1}}$

Figure S8 The calculation formulas
